# Why science needs art

**DOI:** 10.3389/fbinf.2025.1708311

**Published:** 2025-10-24

**Authors:** Giulia Ghisleni, Christian Stolte, Megan Gozzard, Lea Von Soosten, Antonia Bruno

**Affiliations:** 1 Department of Biotechnology and Biosciences, University of Milano-Bicocca, Milan, Italy; 2 Christian Stolte Design, Islesboro, ME, United States; 3 Wellcome Sanger Institute, Hinxton, United Kingdom; 4 University Medical Center Hamburg-Eppendorf (UKE), Hamburg, Germany

**Keywords:** art-science integration, creative epistemology, visual arts in science education, interdisciplinary research, science communication

## Abstract

This perspective paper examines the profound cognitive and methodological parallels between scientific and artistic research, challenging the traditional distinction between the two domains. While science and art use different languages, both emerge from the human drive for creativity and understanding. We argue that scientific inquiry, often presented as strictly objective and methodical, inherently shares with art the need for imagination, flexibility, and interpretative thinking. Drawing on neuroscience, education, design theory, and the visual arts, we highlight how artistic practices, particularly in the visual arts, can enhance scientific learning, innovation, and public engagement. We advocate integrating art into scientific training and research to foster a more creative and inclusive epistemology. Through examples in microbiology, education, and data visualization, we show how the arts can support deeper understanding, cross-disciplinary collaboration, and more effective science communication. Ultimately, we call for a shift toward a more integrated approach that embraces the complementary strengths of both art and science in advancing knowledge and societal impact.

## Introduction

In 1902, Georges Méliès launched what would later be recognized as the first science fiction film, sending two astronomers in a bullet-shaped spacecraft into the eye of the Moon. Before him, H.G. Wells and Jules Verne had described this “science-fiction” journey in their novels, while Giacomo Leopardi addressed the Moon in one of his lyrical poems in the Canti. Decades later, in 1969, Neil Armstrong and Buzz Aldrin set foot on the lunar surface for the first time. Could such a scientific milestone have been achieved without the prior imaginative groundwork laid by art?

Art and science are languages that use different means, yet both are rooted in the same human need for creativity. Both science and art sometimes deliberately set aside strict facts in order to explore deeper truths: science through hypotheses or idealized models; art through fiction and the exploration of human experience, emotion, and meaning. New ideas emerge from the creative process of imagining what could be, rather than limiting inquiry to what is (Elgin, 1993; Eno and Adriaanse, 2025). While this interplay may be intuitive to educators and neuroscientists, it is frequently overlooked by highly specialized scientists and artists. The risk is clear: scientists lacking creativity and artists lacking a sense of reality, ultimately missing the opportunity for interdisciplinary enrichment.

In this perspective paper, we want to question how science is perceived by scientists themselves. We explore the shared cognitive foundations and creative processes of art and science, advocate for their integration in scientific research and education, and examine how visual arts can enhance scientific learning, innovation, and public engagement.

## Toward a creative epistemology

Hannula et al. (2014) define artistic research as a process that involves technical proficiency, conceptual thinking, and creativity, with the aim of producing novel academic contributions and communicating effectively with both peers and the public (Hannula et al., 2014). But how distinct is artistic research from scientific research? The answer is nuanced. While differences exist, strictly separating the two in the name of creativity-driven discoveries risks overlooking their value in both disciplines (Nisbet, 2017). Scientific research is often distinguished by its reliance on scientific methods (plural, not singular) that establish its robustness, reproducibility, and significance (Hannula et al., 2014). Textbooks and school science education frequently present a reductive view of the scientific method as a rigid, step-by-step process involving observation, hypothesis formation, experimentation, and conclusion (Blachowicz, 2009). This oversimplified narrative shapes how students and educators perceive science (Osborne et al., 2003), often distancing learners from the dynamic nature of real research. In reality, scientific methods are diverse and adaptive. While upholding principles like integrity and reproducibility, methodology must evolve alongside emerging technologies and paradigms (Hepburn and Andersen, 2015).

Historical breakthroughs have repeatedly challenged fixed methodological norms. Paul Feyerabend famously argued in Against Method that there are no rules that should not be broken to assure scientific development, provocatively suggesting that “anything goes” if it advances knowledge (Feyerabend, 2020). His stance calls for a shift from control to creative surrender, a willingness to break boundaries, embrace uncertainty, and imagine beyond what is currently considered possible.

**FIGURE 1:**
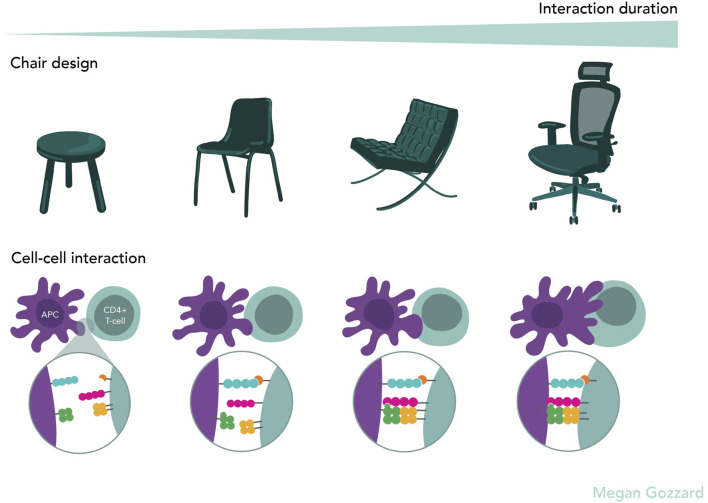
Chairs as a design analogy for the evolution of optimised cell–cell interactions © 2025 by Megan Gozzard is licensed under CC BY-SA 4.0. Progression of chair design illustrates how interactions evolve over time towards increased stability, functionality, and efficiency, analogous to the way cell–cell interactions, such as T cell–APC, develop into stable and highly specialised structures.

If the methodological foundation that defines science is so fluid, then the line between scientific and artistic research becomes increasingly blurred. Research design is intended as a process of building understanding (Xanthoudaki and Blanton, 2021), a definition equally applicable to both art and science. Bruno Munari, an Italian artist, designer, and inventor of the 20th century, extensively described the designer’s method. This, similar to what was discussed above in relation to scientific methods, is adaptable and constantly evolving, aimed at the continuous improvement of the method itself and moving from problem to solution. It involves studying already published research, collecting and analyzing data, applying creativity to generate a further level of understanding, experimentation, and the development of a model (Munari, 2018). Moreover, the similarities extend to outcomes. The creation of a design object is guided by the awareness that structure is translatable to function (Munari and Creagh, 2008). This principle echoes across molecular biology, chemical and medical research, and engineering, where form and function are deeply interlinked (Kohn et al., 2018) (Figure 1).

To foster innovation, scientific training must acknowledge and embrace the creative mechanisms characteristic of artistic research. These include the formulation of questions that involve human roles in the environment, brilliant hypotheses, and innovative experiments, guiding the development of novel, interesting epistemological perspectives (Tyler and Likova, 2012). Below, we will discuss how understanding science can be significantly enhanced through cross-cognitive learning and the integration of creative, visual art perspectives.

## Lessons from visual arts for scientific learning

Learning in the visual arts engages a complex interplay of perceptual, cognitive, and motor functions, reflecting a shared neural foundation that supports cross-cognitive transfer and creativity. A key mental trait in the creative process is the ability to tolerate ambiguity and uncertainty, an essential aspect not only of art but also of scientific inquiry, which often involves navigating contradictions and unresolved questions (Tyler and Likova, 2012). Despite this, science education in schools often emphasizes the memorization of established facts, resulting in a science deprived of its creativity (Xanthoudaki and Blanton, 2021). In contrast, visual arts education provides cognitive skills, such as observation as an advancement of merely looking, being critical of others’ work in relation to standards, envisioning the next steps, expressing ideas and meanings, constructively exploring unknown fields, learning to use tools and conventions, learning history and current practices while transferring this knowledge to others and the society, and embracing problems and persist (Sheridan et al., 2022). Reading this skill list out of context, one could think that these are the must-have qualities of a good scientific researcher.

The subjective nature of visual arts education, often perceived as mutually exclusive with science, can actually enrich scientific learning. While textbooks usually frame problems as closed-ended with a single correct answer, authentic research involves open-ended challenges, such as designing instruments or developing new methods, where outcomes are shaped by subjectivity (Xanthoudaki and Blanton, 2021). Moreover, emotions sustain motivation for learning (Oecd, 2010; Oecd, 2018) and are inevitably part of the cognitive process, even if often regarded as external to rationality (Kozlov, 2023). Science is among the subjects that provoke the highest levels of student anxiety, with significant impacts on learning outcomes. At the same time, however, enjoyment of science is closely tied to students’ perception of its personal relevance, and feelings of confusion can spark the desire to know more (Pekrun and Linnenbrink-Garcia, 2014). Curiosity and wonder are powerful drivers of scientific motivation, and the pleasure of discovery has often been compared to the feeling of a creative artist upon completing a work. Emotions are thus inseparable from reasoning, understanding, learning, and creativity (Kozlov, 2023), and constitute an irreplaceable contribution to science.

Creativity is one of the pillars for deeper learning in all disciplines. It allows the transfer of the acquired knowledge (mastery) into an act of synthesis, being guided by one’s identity and subjectivity (Mehta and Fine, 2015). Creativity fosters understanding, which is different from learning. Understanding means being able to grasp the meaning, context, and implications of knowledge (Elgin, 1993).

Recent educational innovations have embraced these principles through Visual Art-based STEAM (VA-STEAM) educational approaches, which integrate visual arts into the conventional STEM curriculum. Students’ benefits include improvement of causal reasoning, cultivation of observation, association, comparison, and critical thinking, along with the capability of applying creative thinking and multidisciplinary knowledge in design-based and problem-based projects (Zhang and Jia, 2024; Milkova et al., 2013; Aghasafari, 2024; Wiedemeier and Kim, 2025). Arts are a means to learn how to learn (Cuncliffe, 1999) and to learn to be creative (Roege and Kyung, 2013).

## Bridging science and society through art

An artistic researcher faces the need to communicate their research to the public. Modern design and performance art reflect this need by involving the public as users, spectators, or participants (Munari and Creagh, 2008). Scientists face the same need, especially when seeking real societal impact (Bruno et al., 2024). While scientific outputs are often encoded in technical language, statistical models, and abstract representations, art can distil key concepts and resonate emotionally and experientially. Human memory favours stories, and scientific communication can benefit from chronological and logical narratives. These make scientific stories more engaging, memorable, and ultimately more impactful (Medved and Keith, 2000). Some forms of art and science are closer to everyday life than others. A scientist developing anti-cancer drugs translates their research most directly into patient benefit, just as a designer creating a chair closely evaluates human use. By contrast, abstract art and fundamental scientific research may appear more distant from daily experience. Yet these foundational pursuits, such as developing genomic sequencing technologies or creating the optimal material for an ergonomic chair, act as materials and scaffolding that ultimately enable transformative breakthroughs. Without fundamental science, art, and design, applied forms would lack depth, innovation, and vision. The distance from “user” demands communication channels connecting them to practical and social realities (Većkalov et al., 2025). Museums, galleries, classrooms, and conferences facilitate engagement and translation, allowing artists and scientists to share their work integrating different languages.

**FIGURE 2:**
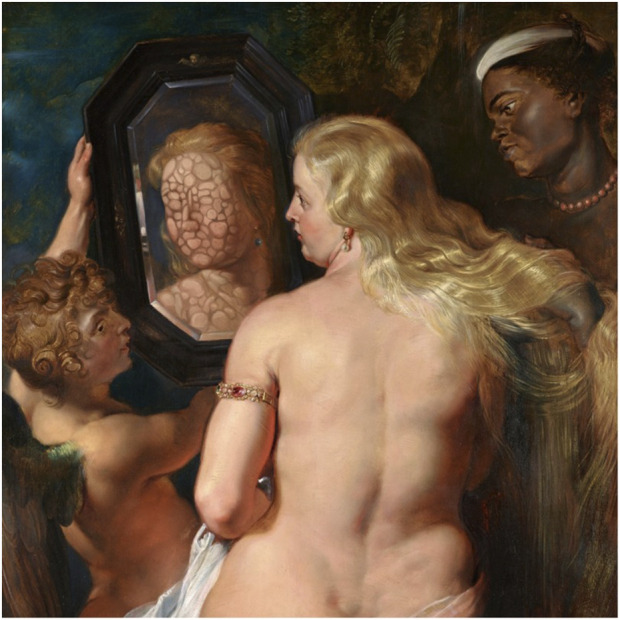
Reflection of the Invisible: Unveiling the Symbiotic Self. Art has long served as a reflection of the human experience, a mirror held up to reality. In this figure, Venus, gazing into her mirrors, does not see her reflection. Instead of the singular self, the mirror shows the vibrant multitude of symbiotic organisms that form the holobiont. In this example, art serves to communicate the importance of studying humans as holobionts, and not as isolated living beings. - Image derived from “Venus in Front of the Mirror” by Peter Paul Rubens (public domain), modified by Giulia Ghisleni, licensed under CC BY-NC-SA 4.0.

One way visual art complements fundamental science is in microbiology, where exhibitions and initiatives have been shown to educate and correct misconceptions about microorganisms, crossing the limits of working on something that is not only invisible but often mistakenly perceived as negligible (Figure 2) (Sangweme et al., 2020; Parks and White, 2021). The European Federation of Microbiology Societies (FEMS) organizes an annual ‘Microbes and Art’ competition and the MicrobiologyInArt blog, aiming to promote compelling portrayals of microorganisms while countering unrealistic representations (Fems, 2025). The Agar Art Contest, organized by the American Society for Microbiology (ASM), invites participants worldwide to create artworks with living bacteria on petri dishes, thereby becoming familiar with culturing methods and with the actual appearance of bacteria. When integrated into undergraduate curricula, this initiative has been shown to inspire creativity and design thinking, while promoting social learning and a deeper understanding of foundational microbiology (ASM.Org, 2018; Sangweme et al., 2020). This format inspired numerous Microbial Art Workshops in Ecuador and living-art exhibitions in New Zealand and the United States. Other contemporary art forms, such as photography, microorganism-colonized sculpture, and dance, were also employed to bridge microbiology and social issues through various initiatives in Mexico and other parts of the world, highlighting the significance of bacteria and, in particular, extremophiles (Rodríguez et al., 2025a; Rodríguez et al., 2025b). Institutions have also embraced this approach: ARTIS-Micropia in Amsterdam, the world’s first museum dedicated to microbes, showcases over 40 species of living microorganisms in an accessible, interactive format (ARTIS, 2025), while the Triennale 24th International Exhibition Inequalities (Milan) presented a microbiome-oriented perspective on architectural history, designed by microbiologists and architects and highlighting the need to design environments that foster healthy interactions with microbial life (Triennale Milano, 2025). Other creative formats documenting scientific progress include graphic storytelling, such as a recent graphic novel narrating the underrepresented story of Fanny Angelina Hesse (1850–1934), who pioneered the use of agar as a growth medium for microbes and thereby transformed microbiology (Fanny-Hesse-Graphic-Novel, 2025), and several mainstream movies that interweave narrative with a realistic portrayal of what microbiologists do and how microorganisms impact human life (Sánchez-Angulo, 2023).

Beyond microbiology and more generally in relation to science, the Visualising Biological Data (VIZBI) conference (Vizbi, 2025), where the ideas developed in this paper were originally discussed, represents the most significant effort by the scientific community to move itself toward a sustainable way of doing integrated science, one that neither isolates nor impoverishes it and allows it to face the challenges of new technologies. Over the past two decades, the amount of data generated has grown exponentially, exemplified by the explosion of sequencing datasets in genomics. The first human genome, completed in 2003, produced around 3 gigabytes of data (Lander et al., 2001; International Human Genome Sequencing Consortium, 2004; Venter et al., 2001); today, advances in sequencing technologies are projected to facilitate the generation of 2–40 exabytes of data within the next decade (Genome, 2025). Making sense of such vast complexity requires not only statistical and computational approaches but also effective visualisation strategies. Good visualisation allows scientists to identify patterns, communicate findings, and highlight key insights, even for expert audiences. A commonly used strategy in genomics is dimensionality reduction, which compresses high-dimensional data into “principal components” that capture and allow visualisation of the dominant trends, with each principal component describing a decreased proportion of the variation than the one before (International Congress of Genetics and Geerts, 1963). Such representations, which distil complexity into a clear and simple message, make overwhelmingly large datasets legible. Data visualisation artists such as John Burn-Murdoch, columnist and chief data reporter at the Financial Times, exemplify how precision and aesthetics combine to create graphics that are both informative and impactful.

Shapes, colours, and patterns directly influence how data visualizations are interpreted and perceived, and thus designed. For example, the concept of visual hierarchy is based in Gestalt psychological theory and describes how viewers’ attention is guided to key data points, due to the human brain having innate organizing tendencies that structure individual elements according to their size, colour, and position, for example, (Koffka, 2013). Moreover, colour palettes can evoke distinct emotional associations, and these vary across geographical and cultural contexts, an often-overlooked factor in global science communication. While most visualisations remain two-dimensional, there are emerging efforts to generate 3D interpretations of data, allowing interaction through virtual reality headsets to “touch” and “feel” data (Molina et al., 2024; Dwb, 2025).

The overlap between artistic and scientific practice is particularly visible here. Tools developed for scientific data visualisation have been harnessed to create artworks, blurring the boundaries between representation and expression, as in the generative work of Andy Lomas. Others, such as Stefanie Posavec and Giorgia Lupi, explored the poetics of personal data through their Dear Data project, exchanging postcards across the Atlantic to document and translate daily patterns into symbolic form (Lupi and Posavec, 2014).

Visualizations serve as tools to read and communicate data, but also, sometimes, to produce data that could reveal valuable insights for scientists to evaluate the impacts (or misdirected impacts) of their research. Valeria Edelsztein, for example, used children’s drawings to assess how the COVID-19 pandemic influenced their understanding of microorganisms, revealing an exclusively negative perception and highlighting the need to reassess didactic strategies to promote scientifically correct models (Edelsztein, 2024).

For a scientific world increasingly built on large-scale datasets, mastering the art of visualisation and fostering collaborations with artists and designers, for example, having a resident illustrator in scientific teams, is becoming fundamental. These practices are not only essential for advancing research itself but also for ensuring that science remains intelligible, memorable, and meaningful to society.

## A short ethical discussion on art and science integration

Our call for multidisciplinarity does not merely derive from the recognition of the mutual benefits that art and science gain from their integration, but arises from a broader ethical perspective that frames how and why scientists do science. As previously discussed, the pursuit of knowledge and the search for truth are among the strongest motivations driving scientific inquiry (Venville et al., 2013). Researchers aim to translate this epistemological drive into scientific advancement and its applications. Scientific ethics provides a framework to ensure that this pursuit aligns with principles including but not limited to honesty, integrity, openness, respect for life, altruism, freedom of research, and fair discussion (Menapace, 2019). Within this framework, art can play an active role in anticipating and reflecting on the ethical implications of scientific progress.

Throughout history, art has often preceded or paralleled major scientific advancements, as in the case of the Moon landing (see Introduction). Similarly, contemporary art continues to expose and question the ethical dimensions of emerging technologies and biotechnological innovation. A clear example of this is the anatomical architectures of the Australian artist Stelarc, who explored the boundaries between biotechnology, the human body, and ethics through his Ear on Arm project (2006), in which a surgically implanted ear on his arm questioned where the line should be drawn between enhancement, experimentation, and necessity (Stelarc Ear On Arm, 2006). Such works catalyze public discourse on the moral consequences of biotechnological interventions.

As previously discussed, engaging in artistic practices connected to science enhances learning, communication, and public involvement. Beyond these educational and communicative values, doing science–art also introduces an additional ethical dimension. It provides opportunities for both creators (to reflect on global scientific issues) and audiences (to consider or reconsider the role of science in society). Bioart, for example, employs biotechnological tools to explore living systems as artistic subjects. This practice guides public engagement with bioethical issues and provokes dialogue on the responsible and informed use of innovation, particularly in fields involving the manipulation of life forms for research or creative purposes (Yetisen et al., 2015; Couture et al., 2017; Fuchs, 2025). From microbial art using living bacteria, as mentioned earlier, to more controversial works such as Alba, the transgenic fluorescent rabbit by Brazilian artist Eduardo Kac, bioart invites us to reconsider the responsibilities shared by artists and scientists alike.

On the more technological end of the spectrum, artists such as Avital Meshi (Meshi, 2024) are exploring the ethical dimensions of humans integrating AI into their day-to-day lives, and ask us to consider the implications of outsourcing moral and practical choices to artificial agents (Avital Meshi, 2025).

Integrating art into science helps researchers uphold their ethical duties through ethical anticipation and societal dialogue, essential components for responsible, sustainable science.

## Conclusion

This perspective highlights the conceptual and methodological parallels between scientific and artistic research, challenging the persistent divide between the two. A change of attitude is needed. Art and science have long been treated as separate realms, but the time has come to reconcile them and embrace the complementarity of these two languages. This will better serve the advancement of understanding and the needs of researchers, whether artistic or scientific, who are increasingly engaged in a form of inquiry that is no longer confined to a single field but is instead multidisciplinary and collaborative.

## Data Availability

The original contributions presented in the study are included in the article/supplementary material, further inquiries can be directed to the corresponding author.

## References

[B1] Aghasafari S. (2024). Creative visual arts and biology processes: examining emergent Bi/Multilingual high schoolers meanings. Eur. J. Arts, Humanit. Soc. Sci. 1 (3), 200–209. 10.59324/ejahss.2024.1(3).18

[B2] ARTIS (2025). What is ARTIS-Micropia?. Available online at: https://www.artis.nl/en/artis-micropia/what-is-artis-micropia (Accessed August 26, 2025).

[B3] ASM.Org (2018). Creating art and education with microbes. Available online at: https://asm.org:443/podcasts/microbeworld-video/episodes/creating-art-and-education-with-microbes.

[B4] Blachowicz J. (2009). How science textbooks treat scientific method: a philosopher’s perspective. Br. J. Philosophy Sci. 60, 303–344. 10.1093/bjps/axp011

[B5] Bruno A. Arnoldi I. Barzaghi B. Boffi M. Casiraghi M. Colombo B. (2024). The one health approach in urban ecosystem rehabilitation: an evidence-based framework for designing sustainable cities. iScience 27 (10), 110959. 10.1016/j.isci.2024.110959 39391715 PMC11466616

[B6] Couture V. Bélisle-Pipon J.-C. Cloutier M. Barnabé C. (2017). Merging arts and bioethics: an interdisciplinary experiment in cultural and scientific mediation. Bioethics 31 (8), 616–630. 10.1111/bioe.12391 28901596

[B7] Cuncliffe L. (1999). Learning how to learn, art education and the ‘background. J. Art and Des. Educ. 18 (1), 115–121. 10.1111/1468-5949.00162

[B8] DWB (2025). Virtual reality for data visualisation. Available online at: https://dwbproject.org/virtual-reality-for-data-visualisation/(Accessed September 1, 2025).

[B9] Edelsztein V. (2024). Has the coronavirus pandemic changed students’ conceptions of microorganisms? Evidence from elementary school. Int. J. Sci. Educ. 46 (8), 733–749. 10.1080/09500693.2023.2256459

[B10] Elgin C. Z. (1993). Understanding: art and science. Synthese 95 (1), 13–28. 10.1007/BF01064665

[B11] Eno B. Adriaanse B. (2025). What art does: an unfinished theory. London: Faber and Faber.

[B12] Fanny-Hesse-Graphic-Novel (2025). Fanny angelina Hesse: a graphic novel. Available online at: https://fanny-hesse-graphic-novel.site/(Accessed August 25, 2025).

[B13] Fems (2025). Microbiology in art. Available online at: https://fems-microbiology.org/volunteering/microbiology-in-art/(Accessed August 25, 2025).

[B14] Feyerabend P. (2020). Against method: outline of an anarchistic theory of knowledge. London: Verso Books.

[B15] Fuchs N. (2025). “Creative synergies: the interplay of art, science, and technology in contemporary culture,” in Human-technology interaction (Springer). Available online at: https://link.springer.com/chapter/10.1007/978-3-031-78357-9_14.

[B16] Genome (2025). Genomic data science fact sheet. Available online at: https://www.genome.gov/about-genomics/fact-sheets/Genomic-Data-Science (Accessed September 1, 2025).

[B17] Hannula M. Suoranta J. Vadén T. (2014). Artistic research methodology. Peter Lang. U. S. 10.3726/978-1-4539-1308-6

[B18] Hepburn B. Andersen H. (2015). Scientific method. Available online at: https://plato.stanford.edu/entries/scientific-method/?utm_source=webtekno#DisSciMet.

[B19] International Congress of Genetics Geerts S. J. (1963). Genetics today: proceedings of the XI international congress of genetics, the hague, the Netherlands, September 1963. With MBLWHOI library. Oxford: Pergamon Press. Available online at: http://archive.org/details/geneticstodaypro01inte.

[B20] International Human Genome Sequencing Consortium (2004). Finishing the euchromatic sequence of the human genome. Nature 431 (7011), 931–945. 10.1038/nature03001 15496913

[B21] Koffka K. (2013). Principles of gestalt psychology. Abingdon: Routledge. 10.4324/9781315009292

[B22] Kohn K. P. Underwood S. M. Cooper M. M. (2018). Connecting structure–property and structure–function relationships across the disciplines of chemistry and biology: exploring student perceptions. CBE—Life Sci. Educ. 17 (2), ar33. 10.1187/cbe.18-01-0004 29786475 PMC5998324

[B23] Kozlov A. (2023). Emotions in scientific practice. Interdiscip. Sci. Rev. 48 (2), 329–348. 10.1080/03080188.2023.2193073

[B24] Lander E. S. Linton L. M. Birren B. Nusbaum C. Zody M. C. Baldwin J. (2001). Initial sequencing and analysis of the human genome. Nature 409 (6822), 860–921. 10.1038/35057062 11237011

[B25] Lupi G. Posavec S. (2014). Dear data - the project. Dear Data. Available online at: http://www.dear-data.com/theproject.

[B26] Medved M. I. Keith O. (2000). Memories and scientific literacy: remembering exhibits from a science centre. Int. J. Sci. Educ. 22 (10), 1117–1132. 10.1080/095006900429475

[B27] Mehta J. Fine S. (2015). The why, what, where, and how of deeper learning in American secondary schools.

[B28] Menapace M. (2019). Scientific ethics: a new approach. Sci. Eng. Ethics 25 (4), 1193–1216. 10.1007/s11948-018-0050-4 29869131

[B29] Meshi A. (2024). GPT-ME: a Human-AI cognitive assemblage. Proc. ACM Comput. Graph. Interact. Tech. 7 (4), 1–8. 10.1145/3664214

[B30] Meshi A. (2025). The AI on my shoulder (2025) - interactive performance with AI agents. Available online at: https://www.avitalmeshi.com/the-ai-on-my-shoulder-2025.html.

[B31] Milkova L. Crossman C. Wiles S. Taylor A. (2013). Engagement and skill development in biology students through analysis of art. CBE—Life Sci. Educ. 12 (4), 687–700. 10.1187/cbe.12-08-0114 24297295 PMC3846519

[B32] Molina E. Kouřil D. Isenberg T. Kozlíková B. Vázquez P.-P. (2024). Virtual reality inspection of chromatin 3D and 2D data. Comput. and Graph. 124 (November), 104059. 10.1016/j.cag.2024.104059

[B33] Munari B. (2018). Da cosa nasce cosa: Appunti per una metodologia progettuale. Bari-Roma: Gius.Laterza and Figli Spa.

[B34] Munari B. Creagh P. (2008). Design as art. Penguin modern classics. Penguin Books.

[B35] Nisbet R. (2017). Sociology as an art form. 2nd ed. New York: Routledge. 10.4324/9781315129990

[B36] OECD (2010). “The nature of learning: using research to inspire practice,” in Educational research and innovation. Editors Dumont H. Istance D. Benavides F. (Paris: OECD). 10.1787/9789264086487-en

[B37] OECD (2018). “Understanding innovative pedagogies: key themes to analyse new approaches to teaching and learning,”, 172. Paris: OECD Education Working Papers. 10.1787/9f843a6e-en

[B38] Osborne J. Simon S. Sue C. (2003). Attitudes towards science: a review of the literature and its implications. Int. J. Sci. Educ. 25 (9), 1049–1079. 10.1080/0950069032000032199

[B39] Parks P. White L. (2021). Foregrounding backgrounds: how scientists conceive art to express the invisible. Sci. Commun. 43 (4), 435–459. 10.1177/10755470211011166

[B40] Pekrun R. Linnenbrink-Garcia L. (2014). International handbook of emotions in education. New York-Abingdon: Routledge.

[B41] Rodríguez Y. Andrés L. Batista-García R. A. (2025a). When science meets creativity: elevating microbiology education with art—two personal experiences. Microb. Biotechnol. 18 (3), e70099. 10.1111/1751-7915.70099 40025646 PMC11872682

[B42] Rodríguez Y. Andrés L. Buela Salazar L. M. Pérez-Llano Y. Gunde-Cimerman N. Batista-García R. A. (2025b). An unexpected facet of extremophiles: their aesthetic potential in artistic expression. Microb. Biotechnol. 18 (10), e70236. 10.1111/1751-7915.70236 41035302 PMC12489178

[B43] Roege G. B. Kyung H. K. (2013). Why we need arts education. Empir. Stud. Arts 31 (2), 121–130. 10.2190/EM.31.2.EOV.1

[B44] Sánchez-Angulo M. (2023). Microbial pathogens in the movies. FEMS Microbiol. Lett. 370 (January), fnad129. 10.1093/femsle/fnad129 38059853 PMC10754150

[B45] Sangweme D. Lampert E. Mcintosh E. (2020). Microbe art can educate and correct misconceptions about microorganisms on JSTOR. Available online at: https://www-jstor-org.unimib.idm.oclc.org/stable/48711087?seq=1.

[B46] Sheridan K. M. Veenema S. Winner E. Hetland L. (2022). Studio thinking 3: the real benefits of visual arts education. New York: Teachers College Press.

[B47] Stelarc Ear On Arm (2006). Stelarc ear on arm. Available online at: http://stelarc.org/_activity-20242.php.

[B48] Triennale Milano (2025). We the bacteria notes toward biotic architecture triennale Milano. Available online at: https://triennale.org/en/events/we-the-bacteria-notes-toward-biotic-architecture (Accessed August 26, 2025).

[B49] Tyler C. W. Likova L. T. (2012). The role of the visual arts in the enhancing the learning process. Front. Hum. Neurosci. 6 (February), 8. 10.3389/fnhum.2012.00008 22347854 PMC3274761

[B50] Većkalov B. Zarzeczna N. Harreveld F. Rutjens B. T. (2025). Psychological distance to science affects science evaluations. J. Soc. Issues 81 (1), e12663. 10.1111/josi.12663

[B51] Venter J. C. Adams M. D. Myers E. W. Li P. W. Mural R. J. Sutton G. G. (2001). The sequence of the human genome. Sci. (New York, N.Y.) 291 (5507), 1304–1351. 10.1126/science.1058040 11181995

[B52] Venville G. Rennie L. Hanbury C. Longnecker N. (2013). Scientists reflect on why they chose to study science. Res. Sci. Educ. 43 (6), 2207–2233. 10.1007/s11165-013-9352-3

[B53] VIZBI (2025). About vizbi. Available online at: https://vizbi.org/About.

[B54] Wiedemeier A. M. D. Kim M. T. (2025). Drawn to BiologyStudent attitudes and perceptions about visual art integration in a biology laboratory. Am. Biol. Teach. 87 (1), 26–31. 10.1525/abt.2025.87.1.26

[B55] Xanthoudaki M. Blanton A. (2021). Creative learning in STEM: towards the design of an approach between theory and reflective practice. J. E-Learning Knowl. Soc. 17 (3), 33–42. 10.20368/1971-8829/1135559

[B56] Yetisen A. K. Davis J. Coskun A. F. Church G. M. Yun S. H. (2015). Bioart. Trends Biotechnol. 33 (12), 724–734. 10.1016/j.tibtech.2015.09.011 26617334

[B57] Zhang C. Jia B. (2024). Enriching STEAM education with visual art: education benefits, teaching examples, and trends. Discov. Educ. 3 (1), 247. 10.1007/s44217-024-00354-w

